# Description of an Ossiform Substance Developed in the Vulvo-Labial Texture; with a Sketch of the Concomitant Symptoms

**Published:** 1817-12

**Authors:** James Kennedy

**Affiliations:** Dunning, Perthshire


### *Description of an Ossiform Substance developed in the Vulvo-labial Texture; with a Sketch of the concomitant Symptoms.* By JAMES KENNEDY, M.D. Dunning, Perthshire.

THE fact of morbid ossification having been developed in various parts of the animal system, has been amply confirmed by the evidence of pathological anatomy. With a view of contributing to the elucidation of this subject, I am induced to communicate an account of this phænonon as it lately occurred in the vulvo-labial texture of a young person, born of healthy and virtuous parents; by whose very intelligent report I am enabled to arrange the particulars of the following case.

J. F. an interesting girl, in her twelfth year, had been unwell for upwards of twenty months, when I was called upon to investigate the origin and nature of her complaint. Towards the close of autumn 1815, while residing with her grandfather in a distant county, she first suffered from the present affection. Instantaneous pain, at that period, attacked the superior portion of the left labium of the vulva. Its occurrence could be traced to no palpable morbific cause; nor did any obvious symptom indicate its approach. It progressively assumed a more severe character. The part became tumefied and livid. Symptomatic fever, meanwhile, was declared; and a large and painful abscess involved the whole labial structure of the affected side. Irritation extended from this to the contiguous parts; and the functions of the bladder were disordered. Some days afterwards, disease, of an analogous character, spread to the inguinal region, and summit of the corresponding thigh; but it subsided without inducing suppuration. The right ankle now became the seat of high inflammation and excruciating pain; and violent erysipelatous symptoms pervaded the whole limb. The abdomen, in a less exquisite degree, displayed the phænomena of morbid action. An abscess was, at last, developed about two inches above the external malleolus of the right extremity. In a short time it burst; discharged, during several weeks, an ichorous sanies; and was then cicatrized by the use an of unctuous application. The other symptoms progressively yielded under the improvement of the general health.

In a fortnight from its formation, the labial abscess burst from an apex pointing anteriorly; and left, on evacuation of its contents, a ragged ulcer. From this, during many weeks, small and irregular quantities of ill-conditioned pus were discharged; and great debility was consequently induced. The febrile symptoms, under these circumstances gradually subsided. Constant head-ache, however, and gastric disorder remained. The bowels were alternately free and constipated; their evacuations occasionally fœtid and discoloured; occasionally natural.

More than once, during the last, and commencement of the present year, inflammation and suppuration alternated with each other in the affected part. Several small ulcers were then successively formed, and healed. About the close of 1816, when the labial discharge was suppressed, a morbid swelling, as large as a walnut, and incessantly painful, arose over the superior sternal bone. It, afterwards, varied considerably in volume; but never assumed a phlegmonous character, nor altogether disappeared. It was smallest when the labial discharge was most abundant; and largest when this was repressed.

June 17th, 1817. The physiognomy of my patient exhibits all the characteristic traits of the lymphatic temperament. Her peison is agreeably proportioned. With a kind and gentle disposition, she is thoughtful beyond her years, and addicted to reading. When unsubdued by the sufferings consequent on her disease, she is sprightly and active. Her digestion is disordered; the alvine functions irregular and defective. The breath is offensive; the tongue, mouth, and fauces, foul and dry. Her sleep is unsound. Feverish heat, accelerated circulation, and acute head-ache, principally seated in the forehead, and somewhat variable in severity, prevail. Frequent perspiration contributes to the exhaustion of her strength. She is weak, but not emaciated. Percussion on the sternal and both costal regions of the thorax, affords no result indicative of internal affection. The abdomen is tumid, tense, and uneasy under pressure.

Some days ago, the left labium of the vulva, on which the ulcerating sore had healed, became painful, and rapidly acquired considerable enlargement. It was livid, elastic, and according to the mother's description, resembled in shape and volume a full-grown female wrist.

On the 15th the tumour burst, when much grumous blood, mixed with purulent matter, escaped. At present (17th) the labium is considerably enlarged; its integuments are thickened, exquisitely vascular, and marked, in several places, with the cicatrices of former sores: it is affected with distressful itching. Puriform sanies exudes from three small sinuous ulcers, situated between the centre and posterior extremity of the labium. Into two of these, the probe penetrates nearly half an inch; into the third, about an inch and a quarter, in a direction slanting downward. The adjacent parts are slightly inflamed, irritable, and itchy. They display no other traces of disease.

*Treatment.* The sores having been well washed with a stimulating lotion, tents were introduced into two of them, and mild dressings superadded. An emetic of ipecacuanha was directed to be forthwith administered; and, in the evening, a cathartic powder composed of rhubarb, submuriate of quicksilver, and an aromatic, followed by a similar dose after an interval of five or six hours. These medicines produced the happiest effect.

Next day, by the use of the probe, I was led to suspect the presence of a foreign body in the diseased labium. By a free incision, therefore, its morbid portion was laid open, amid a profuse flow of unhealthy blood. This hæmorrhage, resulting from the excessive vascularity of the part, rendered examination of its internal condition impracticable. Dry lint was then deposited in the wound, and simple dressing applied over.

Upon removal of the dressings on the following day, I discovered a hard rough substance, lodged in an elastic capsule, and adhering to the bottom of the wound. By means of the common dissecting forceps, it was, with some difficulty, extracted, and proved to be an isolated ossiform concretion, of irregular figure and dimensions. The wound was now cleansed by frequent use of the opiate lotion, and left without any dressing. The labium thus gradually lost its morbid increase of volume; and, in four days, the incision cicatrized. At this time (July 2d) the parts have nearly recovered their natural size and colour. Their inordinate itchiness and irritation are altogether removed.

Freedom of the bowels has, meanwhile, been maintained by the exhibition, every second day, of cathartic powders. Much sordid shreddy fæcal matter, with some lumbricoid and vermicular ascarides, has, by these means, been expelled. The abdomen has become softer, less distended, and less irritable: the head-ache and pain of the sternal tumour have subsided. The latter, however, yet retains the size of a filbert. It imparts an obscure sense of fluctuation, but is confined to the cellular texture; for it moves on the subjacent bone. My patient's strength returns; she acquires plumpness and activity: the energies of her mind revive.

*Description of the ossiform concretion.* The principles and structure of this substance are evidently those peculiar to the osseous system. In form, it possesses an obscure resemblance to an irregular crescent, with horns obtusely styliform. The surfaces are, in some degree, dissimilar: one being rough and cellular, like the spongy structure of long bones; the other more dense, and marked by a transverse depression on one side of its centre. Some of its cells, arranged after the manner of a honey-comb, are transparent. Its convex edge is thin, sharp, jagged; the concave, about one line in thickness, and crenated.

The concretion weighs three grains: its greatest breadth is three-eighths, its greatest length one half, of an inch. Its mean thickness may be estimated at half a line. The presence of such a substance within a soft vascular part must necessarily have proved highly irritant, and been the source of much local pain and disorder.

This preparation, suspended in alcohol, is now transmitted to Dr. Jeffray, for the purpose of being deposited in the Anatomical Museum of the University of Glasgow.

*Postscript.—July the 22d.* The sternal tumour has now become large and very painful. It imparts a more decided sense of fluctuation, but is little discoloured. On being opened, a few drops only of blood issued from the orifice. A roll of lint was, therefore, inserted into the wound, and the parts covered with simple dressing. When the lint was withdrawn on the following day, a considerable quantity of very dense viscid pus came away. The sore was washed with the opiate lotion, and the same treatment repeated on this and on the four subsequent days.

A mass of exuberant granulation, on the 28th, had risen through the wound, and was touched with the nitrate of silver. Next day, adhering to the eschar, a clot of puriform matter was removed with the dressing; and, on being squeezed between the fingers, was found to involve a small rough osseous substance, of the shape and volume of a split pea. This unfortunately was broken and lost. The sore, afterwards, gradually ceased to discharge, and soon cicatrized. At this moment, the part is perfectly sound; the patient healthful and vigorous. Is the lymphatic temperament, when acted upon by disease, favourable to the developement of morbid ossification? K.

*August 8th,* 1817.

